# A sharp decrease in reported non-COVID-19 notifiable infectious diseases during the first wave of the COVID-19 epidemic in the Rotterdam region, the Netherlands: a descriptive study

**DOI:** 10.1186/s12879-022-07209-5

**Published:** 2022-03-03

**Authors:** Babette van Deursen, Margot Hagenaars, Abraham Meima, Liselotte van Asten, Jan Hendrik Richardus, Ewout Fanoy, Helene Voeten

**Affiliations:** 1 Public Health Service Rotterdam-Rijnmond, Rotterdam, The Netherlands; 2 Department of Public Health, Erasmus Medical Center, University Medical Center Rotterdam, Rotterdam, The Netherlands; 3 Center for Epidemiology and Surveillance of Infectious Diseases, National Institute for Public Health and the Environment, Bilthoven, The Netherlands

**Keywords:** Epidemiology, Infectious diseases, Outbreaks, COVID-19, Social distancing

## Abstract

**Background:**

The Public Health Services in the Rotterdam region, the Netherlands, observed a substantial decrease of non-COVID-19 notifiable infectious diseases and institutional outbreaks during the first wave of the COVID-19 epidemic. We describe this change from mid-March to mid-October 2020 by comparing with the pre-COVID-19 situation.

**Methods:**

All cases of notifiable diseases and institutional outbreaks reported to the Public Health Services Rotterdam-Rijnmond between 1st January and mid-October 2020 were included. Seven-day moving averages and cumulative cases were plotted against time and compared to those of 2017–2019. Additionally, Google mobility transit data of the region were plotted, as proxy for social distancing.

**Results:**

Respiratory, gastrointestinal, and travel-related notifiable diseases were reported 65% less often during the first wave of the COVID-19 epidemic than in the same weeks in 2017–2019. Reports of institutional outbreaks were also lower after the initially imposed social distancing measures; however, the numbers rebounded when measures were partially lifted.

**Conclusions:**

Interpersonal distancing and hygiene measures imposed nationally against COVID-19 were in place between mid-March and mid-October, which most likely reduced transmission of other infectious diseases, and may thus have resulted in lower notifications of infectious diseases and outbreaks. This phenomenon opens future study options considering the effect of local outbreak control measures on a wide range of non-COVID-19 diseases. Targeted, tailored, appropriate and acceptable hygiene and distancing measures, specifically for vulnerable groups and institutions, should be devised and their effect investigated.

## Introduction

Reporting to local Public Health Services is mandatory under Dutch law for 51 infectious diseases, as is the reporting of outbreaks by institutions vulnerable for outbreaks, such as kindergartens, (high) schools and healthcare institutions (care or cure) [1]. The Public Health Services Rotterdam-Rijnmond is responsible for the surveillance, control, and prevention of infectious diseases in the Rotterdam region, covering 1.3 million citizens. On 27 February 2020, the first COVID-19 case in the Netherlands was reported. The first nationwide measures were imposed on 12^th^ of March (week 11). The following months, the Public Health Services Rotterdam-Rijnmond observed a substantial decline in non-COVID-19 infectious disease notifications and institutional outbreaks. By then, only few studies had reported on the decline in other infectious diseases during the COVID-19 epidemic. Their focus was mostly on the decreased influenza activity in the Southern hemisphere [2]. The decline in notifications and outbreaks in the Rotterdam region is unprecedented and impacts the current and future public health status of the population as well as the operation of Public Health Services [3]. We here describe the change in notifications of non-COVID-19 infections and institutional outbreaks between mid-March and mid-October 2020 in the Rotterdam region and discuss factors that might have contributed to this decline.

## Methods

All cases of notifiable diseases [4] and institutional outbreaks [5] reported to the Public Health Services Rotterdam-Rijnmond between 1st January and mid-October 2020 were included by date of laboratory confirmation (excluding COVID-19 cases and outbreaks). Data on notifiable diseases were retrieved from Osiris, a database from Center for Epidemiology and Surveillance of Infectious Diseases, The Netherlands. Data on institutional outbreaks were retrieved from HP Zone and MUIZ, which are both controlled by the Public Health Services Rotterdam-Rijnmond. All data were fully anonymized before we accessed them.

The infectious diseases were grouped by type of disease. In this study, we focused on respiratory, gastrointestinal and travel-related diseases. Respiratory diseases included e.g. pertussis, mumps and diphtheria. Gastrointestinal diseases included e.g. norovirus disease, Hepatitis A and Shigellosis. Malaria, Zika, typhoid fever, cholera, yellow fever and paratyphus were categorized as travel-related diseases. Seven-day moving average and cumulative cases were plotted against time and compared to those of 2017–2019 (average and range of the 3 years). Additionally, Google mobility transit data of the Rotterdam region (available for 14 of 15 municipalities) were plotted as the percentage decrease against the baseline-level of weeks 7 and 8 in 2020 [6]. The Google mobility transit data provide insight into the number of travellers in public transportation, as a proxy for social distancing. In two graphs, we added arrows to indicate the timing of which measures were taken or lifted by the Dutch government. Each arrow indicates a different taken nationwide measure and are described in the corresponding caption. Hygiene measures and 1.5 m interpersonal distancing were implemented from week 11 throughout the total study period.

## Results

### Reported notifications of non-COVID-19 infectious diseases

**Fig. 1:**
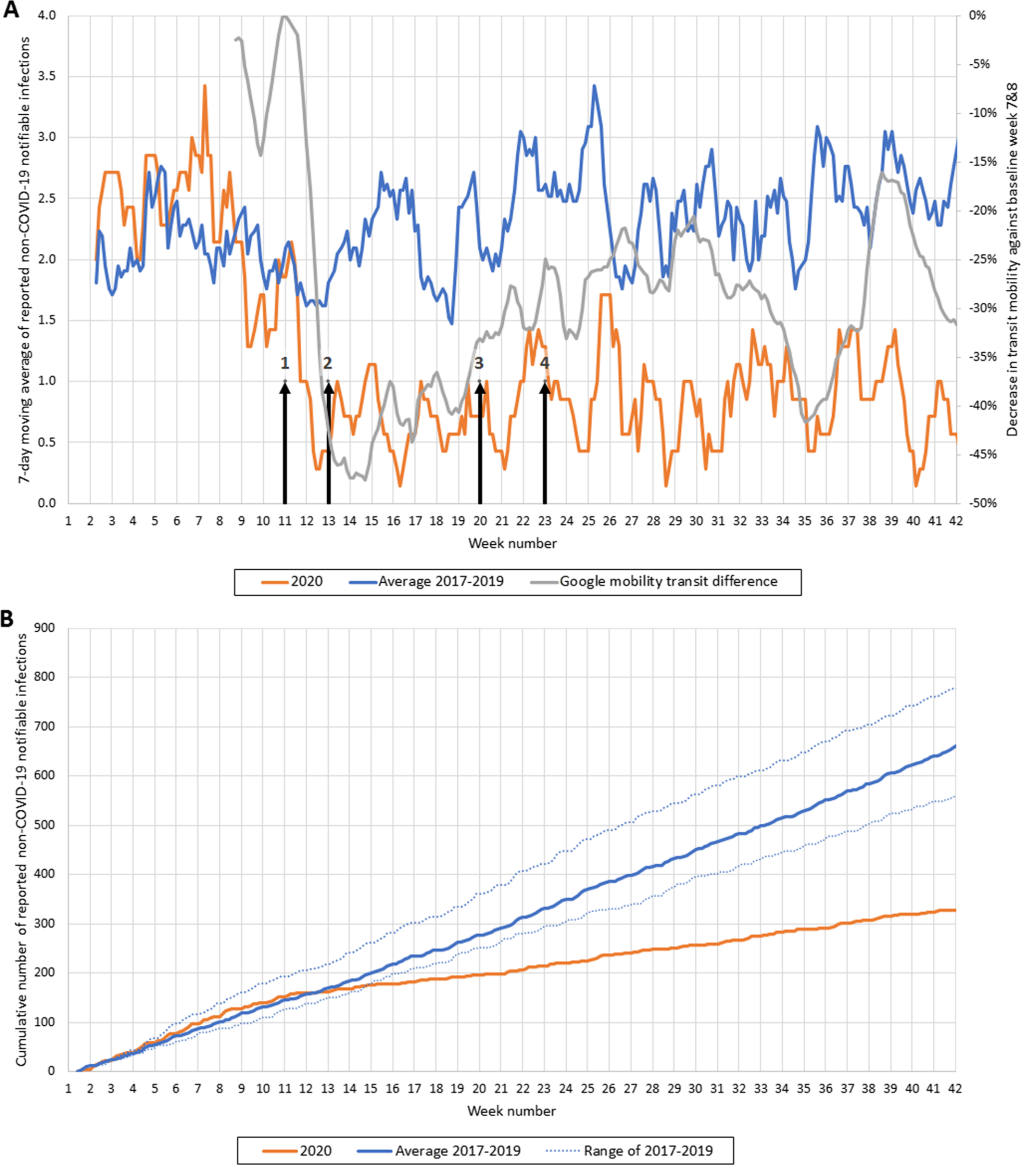
**A** Reported notifications of non-COVID-19 infectious diseases (7-day moving average) in 2020 and 2017–2019. 7-day moving average of notifications of infectious diseases in 2020 (orange line) and the average of 2017–2019 (blue line) and Google mobility transit data given as the percentage decrease compared to 14–24 February 2020 (grey line). The arrows indicate the timing of measures taken by the Dutch government: (1) advised to work from home as much as possible, schools and bars/restaurants were closed; (2) non-medical contact-based professions not allowed to be carried out; (3) elementary schools reopened and contact-based professions allowed to be carried out again; (4) restaurants/bars, museums and high schools reopened. Hygiene measures and 1.5 m interpersonal distancing were implemented from week 11 throughout the total study period. **B** Cumulative number of non-COVID-19 infectious disease notifications in 2020 and 2017–2019. Cumulative number of notifications of infectious diseases in 2020 (orange line); and the average cumulative reports of 2017–2019 (blue line) and the range of 2017–2019 (blue dotted lines)

The total number of notifications of all notifiable diseases combined dropped sharply when the first nationwide measures were imposed (arrow 1, week 11, Fig. 1A), to levels four times lower than the 2017–2019 average. Notifications did not decrease further when additional measures were imposed two weeks later (arrow 2, week 13). The decrease in notifications corresponds with the Google mobility transit data, which also show an initial sharp mobility decline from week 11 to 13 and then a slow increase over time. No increase in notifications was observed after reopening restaurants, bars, museums, and high schools (week 23, arrow 4), not even when mobility temporarily increased in weeks 26–31 and 38–41. Some social distancing measures, however, remained in place throughout the study period, i.e. no hand shaking and keeping physical distance. The initial sharp drop in reported notifications after week 11 is also reflected in the cumulative trend (orange line), which bends downwards from week 11 onwards (Fig. 1B). The blue line reflects the mean of 2017–2019 and the blue dotted lines the range (2017 being the minimum and 2019 the maximum). On average, 660 cumulative cases of infectious diseases were yearly reported from week 1 to 42 in 2017–2019, while in 2020, 328 cumulative cases were reported (50% of 660) of which almost half (n = 146) were reported before the start of the COVID-19 epidemic. This decline could be explained by low numbers in all three separate disease groups (reported non-COVID-19 respiratory, gastrointestinal, and travel-related diseases), as further described below. To compare with COVID-19: the Public Health Service Rotterdam-Rijnmond received approximately 47,000 notifications of COVID-19 infections during March 2020–October 2020.

**Fig. 2:**
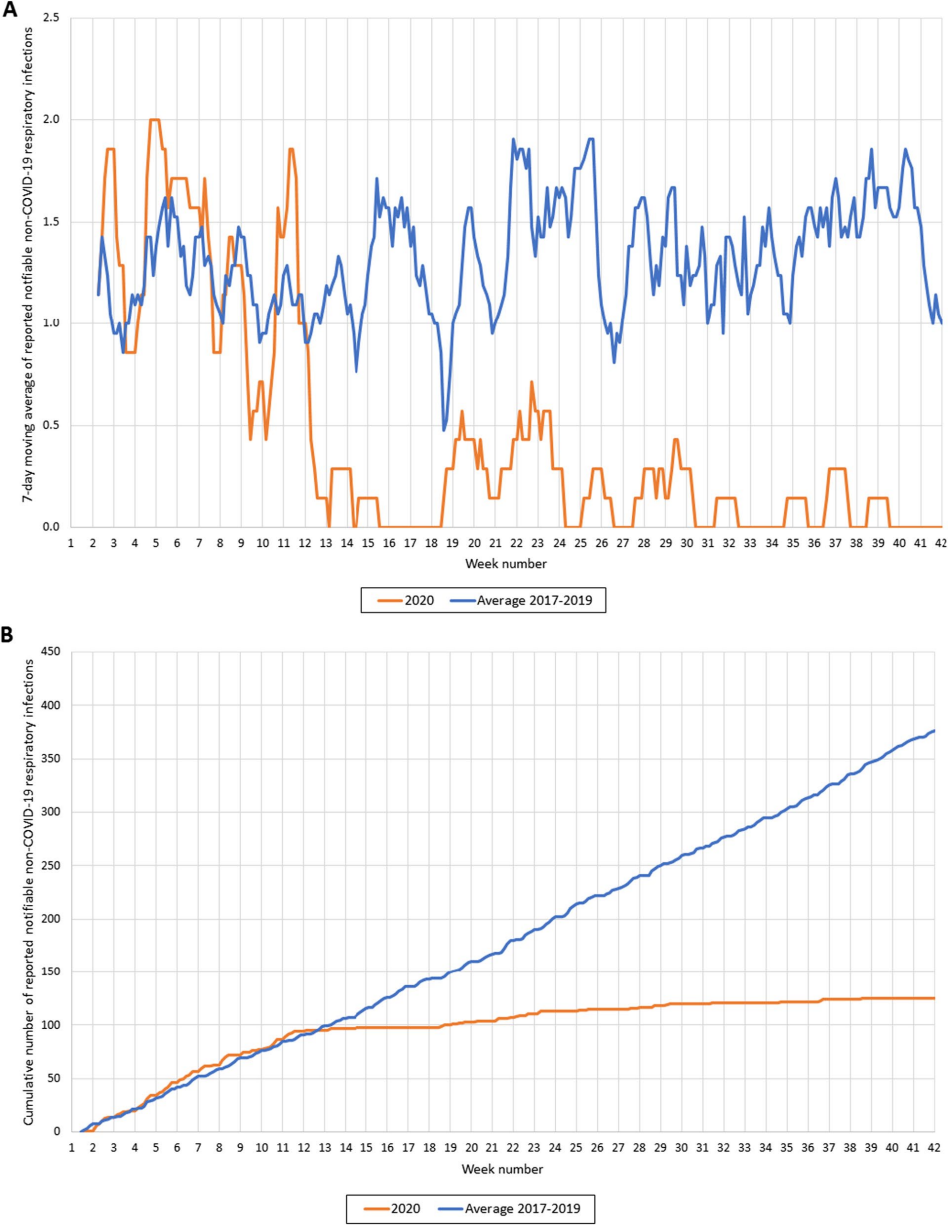
**A** Reported notifications of non-COVID-19 respiratory infectious diseases (7-day moving average) in 2020 and 2017–2019. Notifications of respiratory infectious diseases (7-day moving average) in 2020 (orange line) and the average number of notifications of respiratory infectious diseases in 2017–2019 (blue line). **B** Cumulative number of non-COVID-19 respiratory infectious disease notifications in 2020 and 2017–2019. Cumulative number of respiratory infectious diseases notifications in 2020 (orange line) compared to the average of 2017–2019 (blue line)

The number of reported non-COVID-19 respiratory infections dropped substantially after week 11 (Fig. 2A). A slight increase was observed after week 18, but the number of notifications generally remained low over time. In 2017–2019, 90% of respiratory notifications was due to pertussis; this proportion remained the same in 2020 but numbers were much lower. In the years 2017–2019, 376 respiratory infections were on average reported from week 1 to 42, while in 2020, only 125 respiratory infections were reported (33% of 376) (Fig. 2B). Most of these infections were reported before the first measures in week 11 (n = 85, 68%); the cumulative curve almost flattened afterwards.

**Fig. 3:**
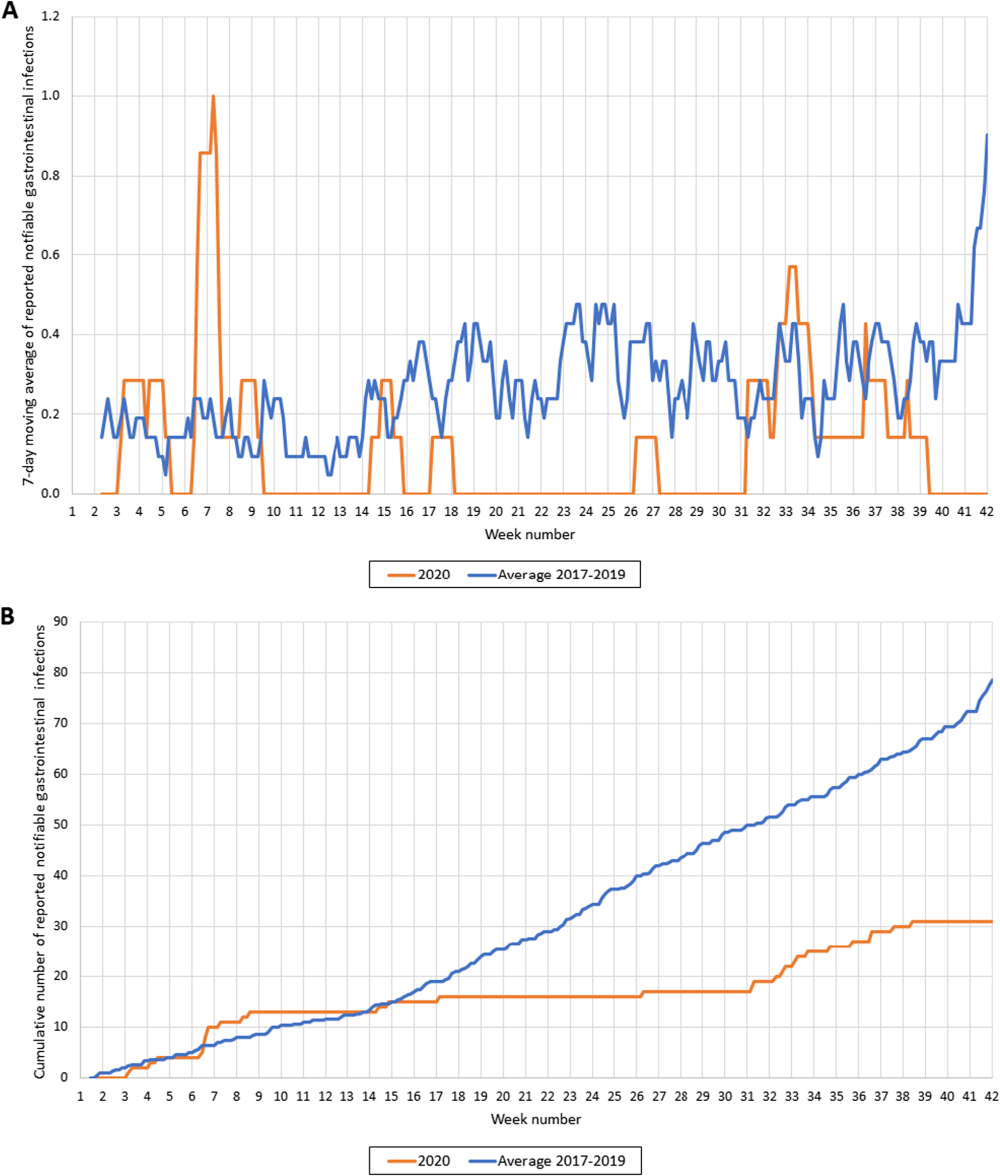
**A** Reported notifications of gastrointestinal infectious diseases (7-day moving average) in 2020 and 2017–2019. Notifications of gastrointestinal infectious diseases (7-day moving average) in 2020 (orange line) and the average number of notifications of gastrointestinal infectious diseases in 2017–2019 (blue line). **B** Cumulative number of gastrointestinal infectious disease notifications in 2020 and 2017–2019. Cumulative number of reported notifiable gastrointestinal infectious diseases in 2020 (orange line) and the average of 2017–2019 (blue line)

Gastrointestinal disease notifications were reported less often than the average of 2017–2019 and remained lower (often at zero weekly cases) for most of the study period, except for weeks 31–34 when the notifications shortly rebounded to 2017 – 2019 levels (Fig. 3A). In the years 2017–2019, on average 79 cases of gastrointestinal diseases were reported from week 1 to 42, while only 31 cases (39% of 79) were reported in 2020 (Fig. 3B). Before the first measures in week 11, most of these gastrointestinal notifications were reported (n = 13, 42%).

In 2020, 10 cases of travel-related disease notifications were reported, versus 24 yearly cases on average in 2017–2019. Notifications of other notifiable infectious diseases, i.e. (chronic) hepatitis, were not noticeably less reported than in preceding years. Non-COVID-19 respiratory, gastrointestinal, and travel-related diseases were together notified 65% less often during the first wave of the COVID-19 epidemic than in the same weeks in 2017–2019.

### Reported non-COVID-19 institutional outbreaks

**Fig. 4:**
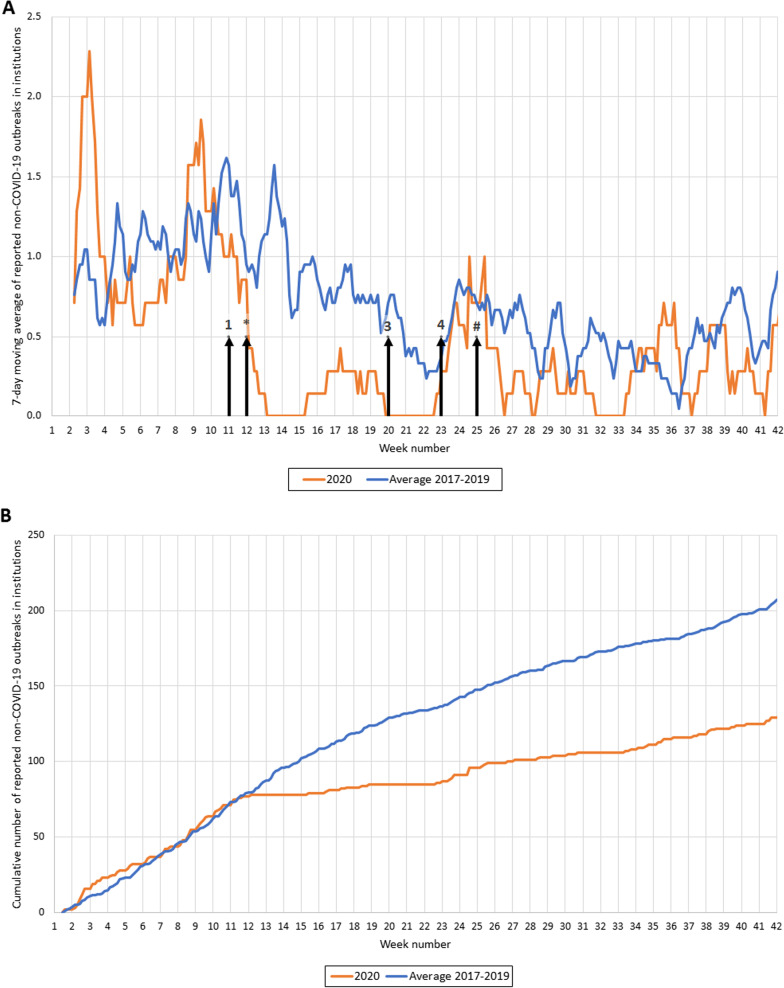
**A** Reported non-COVID-19 institutional outbreaks (7-day moving average) in 2020 and 2017–2019. 7-day moving average of reported outbreaks in institutions in 2020 (orange line) and the average of reported outbreaks in 2017–2019 (blue line). The arrows indicate the timing of the measures taken by the Dutch government: (1) advised to work from home as much as possible, schools and bars/restaurants were closed; (*) no visitors allowed in the nursing homes; (3) elementary schools reopened and contact-based professions allowed to be carried out again; (4) restaurants/bars, museums and high schools reopened; (#) visitors were allowed in nursing homes. Hygiene measures and 1.5 m interpersonal distancing were implemented from week 11 throughout the total study period. **B** Cumulative number of reported non-COVID-19 institutional outbreaks in 2020 and 2017–2019. Cumulative number of reported outbreaks in institutions from 2020 compared to the average in 2017–2019

The number of reported institutional outbreaks was slightly elevated in weeks 8 and 9 of 2020 (the first nationally reported COVID-19 case was at the end of week 9), and subsequently declined and dropped far below the 2017–2019 levels after the imposed measures in week 11 and 12 (Fig. 4A). After reopening high schools (week 23), the number of reported institutional outbreaks rebounded but not quite to 2017–2019 average levels. In the years 2017–2019, on average 207 institutional outbreaks were reported from week 1 to 42, while in 2020, 129 outbreaks were reported (62% of 207) (Fig. 4B). Comparatively, there were approximately 215 COVID-19 outbreaks reported during the same period in 2020. The reported non-COVID-19 outbreaks in 2017–2019 were mostly occurring in kindergartens (38%) followed by healthcare institutions (36%), while in 2020, more than half of the outbreaks were reported by kindergartens and 26% by healthcare institutions. No substantial difference was seen among (high) schools (13% in 2017–2019 versus 17% in 2020).

## Discussion

Reports of non-COVID-19 respiratory, gastrointestinal, and travel-related notifiable diseases showed a 65% reduction between mid-March and mid-October 2020 compared to the previous 3 years. This could be explained by the decline in contacts which we observed in the sharp decrease of the Google mobility transit data of the Rotterdam region after the first imposed nationwide measures. The decline in contacts after the imposed measures was also recently described in another Dutch study [7]. Furthermore, other countries observed a decrease in non-COVID-19 respiratory infections during the COVID-19 pandemic as well, especially in influenza notifications [2, 8–10]. These studies suggested that the decrease in influenza activity and other non-COVID-19 respiratory infections was related to the imposed social distancing measures, since these measures also limit the transmission of other pathogenic microbes. While some measures were relaxed by week 20 and 23, the hygiene and physical distancing measures always remained in place in the Netherlands, which could explain the low reported number of respiratory infections during the rest of the year. Furthermore, travelling between countries during the COVID-19 epidemic was highly discouraged, most likely reducing the notifications of travel-related diseases. At the beginning of the study, literature on the decline of other infectious diseases during the epidemic, especially on gastrointestinal diseases, was sparse. Gastrointestinal diseases are most likely transmitted by food or faecal-oral route, implying that the observed decline may be explained by less human-to-human contact and better hand hygiene. Recently, it was reported that the number of foodborne outbreaks in Europe also decreased in 2020 compared to 2019 [11, 12].

Underreporting and underdiagnosing due to change in health care consumption can also be a reason for the observed decline in the 2020 notifications. General practitioners in the Netherlands, who are the first point of contact for health care, reported a low number of consultations at the beginning of the COVID-19 epidemic [13]. Additionally, laboratories limited their test capacity for other viruses to increase capacity for SARS-Cov-2 testing [14], thus potentially decreasing detections of other infectious diseases.

Non-COVID-outbreaks in institutions were barely reported during the first wave of the COVID-19 epidemic. However, after allowing visitors into the nursing homes and reopening schools, the number of outbreaks increased. Previously, school closure was also observed to limit influenza transmission in France [15]. Our observations seem to confirm that by closing nursing homes and schools, transmission and outbreaks of other infectious diseases were indeed reduced. Reopening brought non-COVID-19 institutional outbreaks back roughly to historical levels, despite continuation of hygiene measures and physical distancing.

While the decline in notifications is clearly obvious from the descriptive exploration, our study has a few limitations. We used Google mobility transit data as a proxy for social distancing. Data on other imposed control measures (e.g. hand hygiene) were lacking. Also, data on health care consumption and the limited laboratory test capacity due to COVID-19 were not available to us and thus a more complex time series analysis could not be considered.

## Conclusions

During the first wave of the COVID-19 outbreak in the Netherlands, the Public Health Services Rotterdam-Rijnmond observed a remarkable 65% reduction in notifiable infectious diseases and 38% reduction in institutional outbreaks compared to the preceding 3 years. While the number of notifications mostly remained low for the remainder of 2020, outbreaks in institutions rebounded to near historical levels when nursing homes reopened for visitors. The timing of the decline suggests that it was related to reduced transmission due to the imposed social distancing measures. Although there is a high community price to pay for some social distancing measures, we did see an impressive effect when implemented nationwide. Therefore, it is worthwhile to explore implementation of socially acceptable, targeted, seasonal, and local or institutional hygiene and social distancing measures to reduce other infectious diseases, such as influenza, and thus prevent or halt future outbreaks or epidemics.

## Data Availability

The used surveillance databases were Osiris, MUIZ and HP Zone. The datasets generated during and/or analysed during the current study are not publicly available but are available from the corresponding author on reasonable request.
